# Overexpression of caldesmon is associated with tumor progression in patients with primary non-muscle-invasive bladder cancer

**DOI:** 10.18632/oncotarget.5458

**Published:** 2015-09-25

**Authors:** Myung-Shin Lee, Jisu Lee, Joo Heon Kim, Won Tae Kim, Wun-Jae Kim, Hanjong Ahn, Jinsung Park

**Affiliations:** ^1^ Department of Microbiology and Immunology, Eulji University School of Medicine, Daejeon, South Korea; ^2^ Department of Pathology, Eulji University School of Medicine, Daejeon, South Korea; ^3^ Department of Urology, College of Medicine, Chungbuk National University, Cheongju, South Korea; ^4^ Department of Urology, Asan Medical Center, University of Ulsan College of Medicine, Seoul, South Korea; ^5^ Department of Urology, Eulji University Hospital, Eulji University School of Medicine, Daejeon, South Korea

**Keywords:** antibody microarray, bladder cancer, biomarkers, caldesmon, tumor progression

## Abstract

The expression and function of caldesmon (CAD) in urothelial bladder carcinoma (BC) have not been reported. Here, we investigated the expression, prognostic value, and potential functional mechanism of CAD in primary non-muscle-invasive bladder cancer (NMIBC). Protein profiling of tissue samples using antibody microarrays showed significantly higher CAD expression in muscle-invasive BC tissues compared with NMIBC tissues. We then validated the CAD expression in BC cells by immunohistochemistry analysis using paraffin-embedded tissue blocks and western blots using BC cell lines. In addition, we examined the expression of CAD variants by reverse transcription-polymerase chain reaction, and confirmed the expression of low-molecular-weight isoforms (L-CAD), specifically encoded by WI-38 L-CAD II (transcript variant 2), in BC cells. Survival analysis in an independent primary NMIBC cohort comprising 132 patients showed that positive CAD expression was significantly associated with poorer prognosis than no CAD expression with regard to recurrence- and progression-free survival (*p* = 0.001 and 0.014, respectively). Multivariate analyses further indicated that positive CAD expression was an independent predictor of progression-free survival (*p* = 0.032; HR = 5.983). Data obtained from *in vitro* silencing and overexpression studies indicated that L-CAD promotes migration and invasiveness of BC cells. Immunofluorescence assays showed dramatic structural changes in the actin cytoskeleton of BC cells after L-CAD overexpression. Our findings collectively suggest that L-CAD overexpression in primary NMIBC is significantly associated with tumor progression and that a possible mechanism for L-CAD's activity is implicated in increased cell motility and invasive characteristics through morphological changes in BC cells.

## INTRODUCTION

Bladder cancer (BC) is the second most common cancer of the genitourinary tract worldwide [1]. Urothelial carcinomas represent more than 90% of BCs and are classified into non-muscle-invasive BC (NMIBC) and muscle-invasive BC according to the depth of invasion. Approximately 75% of patients with BC are diagnosed with NMIBC [2], which is treated with transurethral resection of bladder tumor (TURBT) with/without intravesical therapy. However, despite complete removal of NMIBC by TURBT, the recurrence rate is 15%–90% within 5 years, and the progression rate is 7%–50% [3–5]. Variable recurrence and progression rates reflect the heterogeneity of NMIBC, while identification of prognostic factors is crucial for optimal management of patients with NMIBC.

Currently, high-throughput technology allows us to analyze multiple concurrent molecular alterations; thus, it is widely used to identify BC-related biomarkers [6–8]. Among the high-throughput technology platforms, we previously demonstrated the potential applicability of antibody microarray (AbM) profiling in identifying novel biomarkers in primary NMIBC [9]. In our previous AbM profiling using tissue specimens, caldesmon (CAD) was identified as one of the proteins with significant differential expression between BC tissue and normal urothelial tissue [9].

CAD, encoded by the CALD1 gene, exists as 5 isoforms. The high-molecular-weight CAD protein (H-CAD; 120–150 kDa) is restricted to visceral and vascular smooth muscle cells, whereas the low-molecular-weight isoforms (L-CAD; 70–80 kDa) are present in nonsmooth muscle cells [10]. CAD is a major actomyosin binding protein that is critically implicated in the regulation of the microfilament network, and acts as an important modulator of various cell functions. CAD expression and its role in several solid cancers have been reported in recent studies [11–15]. However, the results are conflicting. For example, CAD expression is associated with poorer prognosis in patients with oral squamous cell carcinoma [11] and colorectal cancer [12], whereas other studies with human colon [13] and breast cancer cell lines [13, 14], and gastric cancer [15] show the function of CAD as a repressor of cancer cell migration or invasion.

To our knowledge, the expression and functional role of CAD in urothelial BC have rarely been investigated to date, although limited numbers of studies have reported CAD expression in bladder tumors of smooth muscle origin, such as myopericytoma, leiomyoma, leiomyosarcoma, and inflammatory myofibroblastic tumors [16–19]. In this study, we investigated CAD expression in BC and normal tissue samples using AbM, and validated its expression in BC cells using immunohistochemistry (IHC) and western blots as well as CAD variants using reverse transcription-polymerase chain reaction (RT-PCR). Furthermore, we assessed its prognostic value in an independent primary NMIBC cohort. Finally, we elucidated a possible mechanism of action of CAD in BC.

## RESULTS

### Differential protein expression identified by AbM profiling

**Figure 1:**
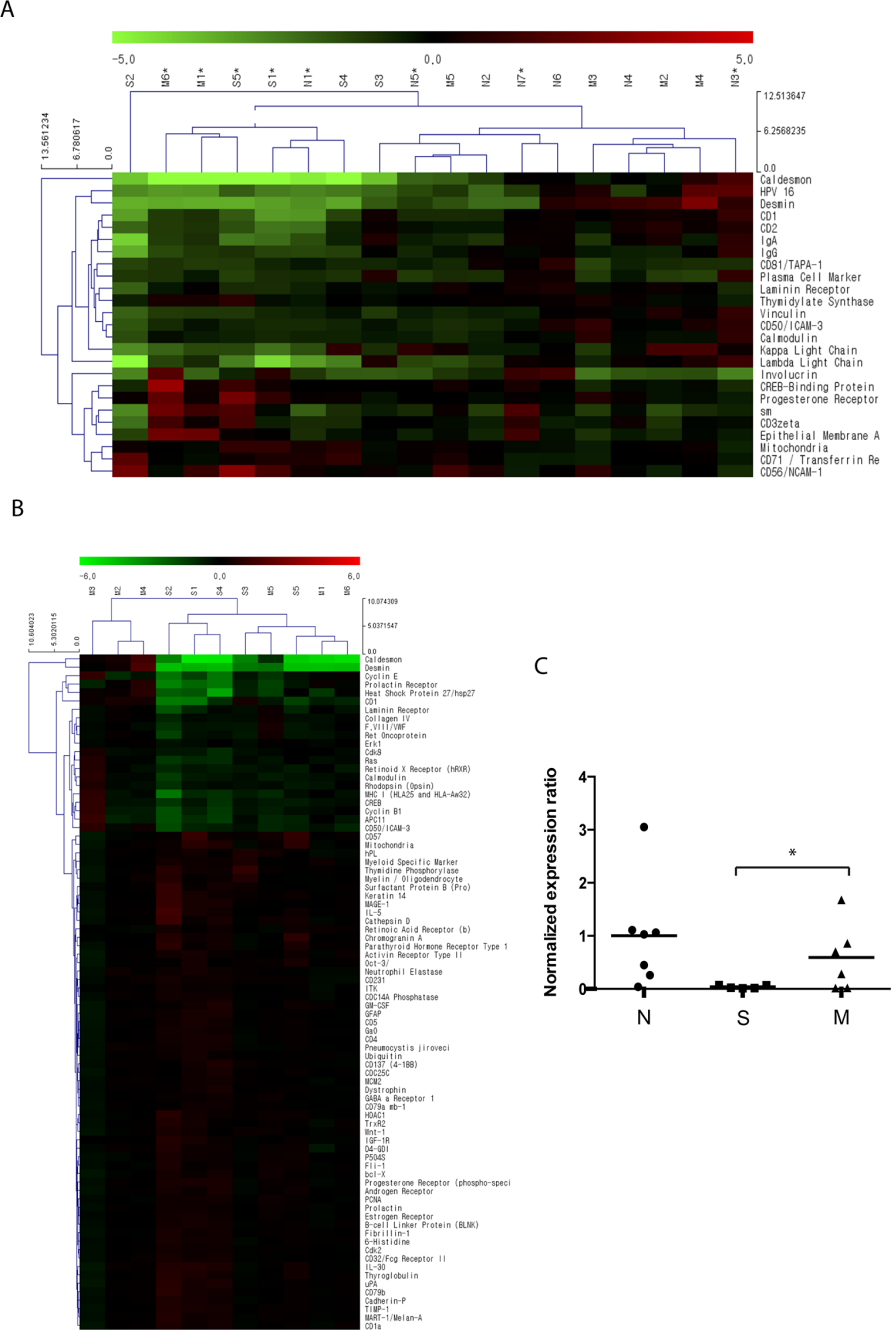
Differential protein expression identified by antibody microarray profiling A total of 11 bladder cancer (BC) tissue samples were acquired from patients who were diagnosed with primary non-muscle-invasive BC (NMIBC) following complete TURBT (*n* = 5, designated as ‘S’) and muscle-invasive BC following radical cystectomy (*n* = 6, designated as ‘M’). A total of 7 normal bladder mucosal tissues (designated as ‘N’) were obtained from the normal bladder mucosa of patients undergoing TURBT (*n* = 3) or from a tissue biobank (*n* = 4, tissues from patients undergoing transurethral resection of the prostate and augmentation cystoplasty). Protein expression in the 18 tissue samples was analyzed using an antibody microarray kit with 656 antibodies. **A.** Hierarchical clustering analysis of protein expression in BC tissues and normal tissues. Proteins shown in the right column are those with *a* > 1.5-fold (or < 0.667) change with *p* values < 0.1. Red indicates higher expression in BC tissues as compared to normal tissues; green indicates lower expression in BC tissues. Further detailed information is accessible through GEO Series accession number GSE69736 (http://www.ncbi.nlm.nih.gov/geo/query/acc.cgi?acc=GSE69736). **B.** Differential protein expression between primary NMIBC and muscle-invasive BC tissues. Proteins shown in the right column are those with *p* values < 0.1. Red indicates higher expression in NMIBC compared to muscle-invasive BC tissues; green indicates lower expression in NMIBC tissues. Expression of caldesmon in muscle-invasive BC tissues was significantly higher than that in NMIBC tissues. **C.** Statistical analysis for normalized expression ratio of caldesmon in the antibody microarray profiling (*p = 0.043).

Differential protein expression between BC tissues and normal urothelium was selected based on the fold change (>1.5-fold or <0.667-fold) and statistical significance (*p* < 0.1) (Figure 1A). Consistent with our previous study that included three paired tissue samples of NMIBC and normal urothelium [9], CAD had significantly lower expression in BC tissues compared to normal bladder mucosal tissues. Of note, the expression of CAD in muscle-invasive BC tissues was significantly higher than that in NMIBC (superficial) tissues (Figure 1B and 1C, *p* = 0.042).

### Expression of CAD in human BC cells

**Figure 2:**
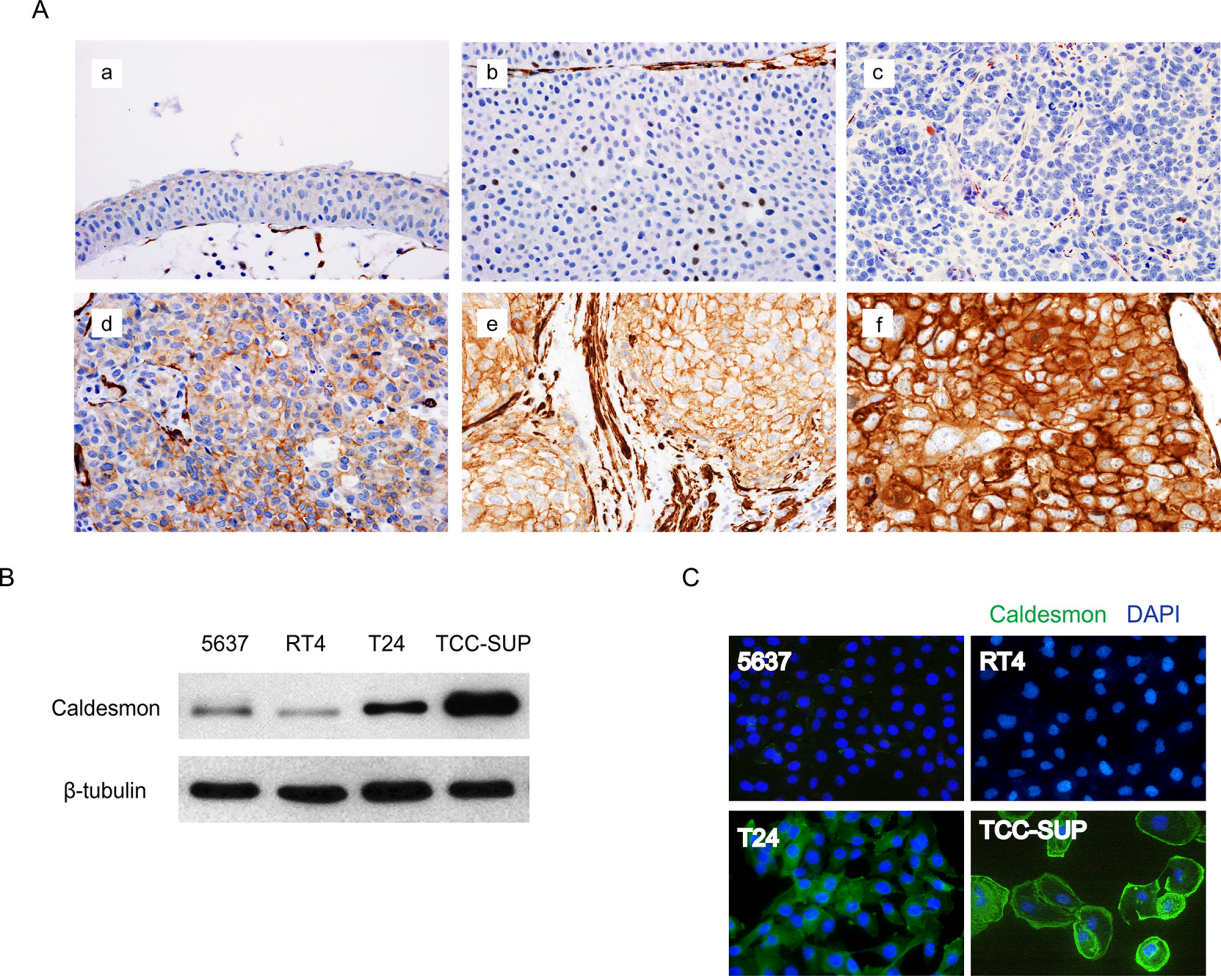
Protein expression of caldesmon (CAD) in human bladder cancer (BC) tissues and cell lines **A.** Representative images of immunohistochemical detection of CAD in human BC tissues (magnification, 400×). a. normal urothelium, b. negative expression in low-grade non-muscle-invasive BC (NMIBC), c. negative expression in high-grade NMIBC, d. mild expression in high-grade NMIBC, e. moderate expression in high-grade NMIBC, f. strong expression in high-grade muscle-invasive BC. **B.** Total CAD protein expression in several BC cell lines was analyzed by western blotting. β-tubulin was used as a calibration control. **C.** Expression of CAD as examined by an immunofluorescence assay. Representative images are shown from 3 independent experiments. Nuclei and target proteins were detected using immunofluorescence staining and examined using a Nikon Eclipse E400 microscope at 400× magnification.

To verify the AbM results, the expression of CAD in BC and normal urothelial cells was examined in human tissue paraffin blocks by IHC (Figure 2A). While CAD was expressed primarily in the cell membrane and cytoplasm of BC cells, its expression was significantly higher in muscle-invasive high-grade BC cells compared with NMIBC cells, consistent with the results of AbM profiling. However, CAD expression was absent or very weak in normal urothelial cells, although normal bladder tissues showed higher CAD expression compared with BC tissues in the AbM. Because AbMs are based on protein extracts from tissues, these inconsistent results between normal bladder tissues and urothelial cells are thought to be likely due to stromal components [9].

Expression of CAD was also investigated in several BC cell lines, including 5637, RT4, T24, and TCC-SUP. While CAD expression was variable depending on the cell line, BC cell lines with higher invasive potential had higher CAD expression in western blot analysis (Figure 2B), consistent with the AbM and IHC results. These significant differences in CAD expression among BC cell lines were also confirmed by an immunofluorescence assay (IFA) (Figure 2C).

### Identification of CAD isoforms in BC

**Figure 3:**
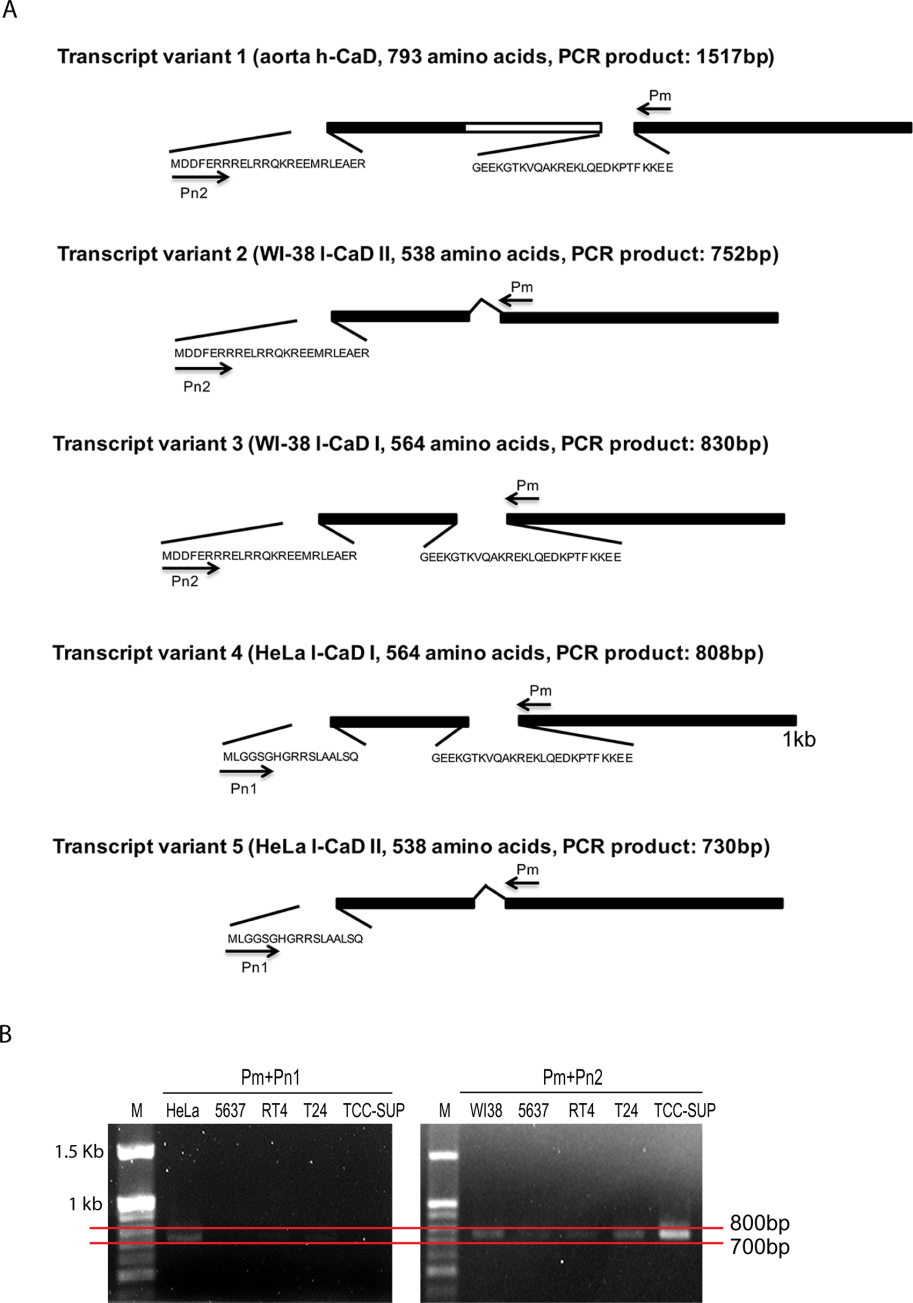
Analysis of the expression of caldesmon (CAD) variants in bladder cancer (BC) cell lines **A.** Schematic summary of the CAD isoforms. The identical sequences in all CAD isoforms and the central repeating domain specific to H-CAD are indicated by solid bars and open bar, respectively. The short amino acid sequences specific to CAD variants are shown. Primers used in PCR analysis are indicated by arrows and the sizes of amplified PCR products in each transcript variant are described. **B.** Agarose gel electrophoresis of CAD RT-PCR products from BC cell lines. RT-PCR was conducted using first-strand cDNA from each BC cell line with the indicated primer set. HeLa S3 (HeLa) and WI-38 (WI38) cells were used as positive controls. M: 100 bp DNA size marker.

Given that five different isoforms of CAD have been identified [10], RT-PCR was performed to characterize CAD expression in BC. Sequence analysis revealed four different isoforms of L-CAD that originated from HeLa S3 and WI38 cells. A schematic summary of CAD transcript variants and amplified fragment sizes using designed primers is shown in Figure 3A. The sense primers Pn1 and Pn2 were designed to anneal specifically to gene fragments encoding the amino terminal sequences of CAD isoforms. Major amplified PCR fragments with primer Pn2 were detected between 700 bp and 800 bp, indicating that the main CAD isoform in BC cells was transcript variant 2 (WI-38 L-CAD II, expected PCR product is 752 bp) (Figure 3B). Primer Pn1 did not produce any amplified fragments in the BC cell lines, and no amplified band was observed at 1.5 kb in any samples.

### Association between immunohistochemical CAD expression and clinicopathological characteristics

To evaluate the relevance of CAD as a clinical biomarker in BC, we analyzed immunohistochemical expression from an independent primary NMIBC cohort comprising 132 patients. Table 1 summarizes the baseline characteristics of a validation cohort. The median age of the patients was 68 (range 28–85) years. The immunohistochemical scores, based on staining area and intensity, were as follows: CAD expression was negative in 43 patients (32.6%), mild in 35 patients (26.5%), moderate in 37 patients (28.0%), and strong in 17 patients (12.9%). Based on these data, CAD expression was dichotomized as negative versus ≥ mild (designated as “positive”), because such grouping showed the most significant survival difference in the Kaplan–Meier analysis. Positive expression of CAD was significantly associated with adverse pathological characteristics, including large tumor size (≥3 cm), lymphovascular invasion, intravesical therapy, higher stage, and grade (Table 2).

**Table 1:** Baseline characteristics of a validation cohort comprising 132 patients with primary non-muscle-invasive bladder cancer

Variables	No. (%)
Gender	
Male	107 (81.1)
Female	25 (18.9)
Tumor size	
<3 cm	94 (71.2)
≥3 cm	38 (28.8)
Multifocality	
Single	100 (75.8)
Multiple	32 (24.2)
Concomitant carcinoma-in-situ	
No	110 (83.3)
Yes	22 (16.7)
Morphology	
Papillary	117 (88.6)
Sessile	15 (11.4)
Lymphovascular invasion	
No	108 (81.8)
Yes	24 (18.2)
Intravesical therapy	
No	71 (53.8)
Yes	61 (46.2)
Tumor stage	
Ta	57 (43.2)
T1	75 (56.8)
Grade	
Low	77 (58.3)
High	55 (41.7)
Recurrence	
No	86 (65.2)
Yes	46 (34.8)
Progression	
No	113 (85.6)
Yes	19 (14.4)

**Table 2:** Association between caldesmon expression and clinicopathological characteristics

Variables	Caldesmon expression*		*p*
	Negative	Positive	
Total no.(%)	43 (32.6)	89 (67.4)	-
Gender (no. [%])			0.176
Male	32 (74.4)	75 (84.3)	
Female	11 (25.6)	14 (15.7)	
Tumor size (no. [%])			0.009
<3 cm	37 (86.0)	57 (64.0)	
≥3 cm	6 (14.0)	32 (36.0)	
Multifocality (no. [%])			0.293
Single	35 (81.4)	65 (73.0)	
Multiple	8 (18.6)	24 (27.0)	
Concomitant carcinoma *in situ* (no. [%])			0.115
No	39 (90.7)	71 (79.8)	
Yes	4 (9.3)	18 (20.2)	
Morphology (no. [%])			0.773
Papillary	39 (90.7)	78 (87.6)	
Sessile	4 (9.3)	11 (12.4)	
Lymphovascular invasion (no. [%])			0.005
No	41 (95.3)	67 (75.3)	
Yes	2 (4.7)	22 (24.7)	
Intravesical therapy (no. [%])			0.010
No	30 (69.8)	41 (46.1)	
Yes	13 (30.2)	48 (53.9)	
T stage (no. [%])			<0.001
Ta	38 (88.4)	19 (21.3)	
T1	5 (11.6)	70 (78.7)	
Grade (no. [%])			<0.001
Low	37 (86.0)	40 (44.9)	
High	6 (14.0)	49 (55.1)	

* The immunohistochemical score was based on both staining area and intensity, and caldesmon expression was negative in 43 patients (32.6%), mild in 35 patients (26.5%), moderate in 37 patients (28.0%), and strong in 17 patients (12.9%). Its expression was dichotomized (negative vs. positive) because such grouping showed the most significant survival difference in the Kaplan–Meier analysis.

### Prognostic value of CAD expression

The median follow-up was 48.4 mo. (mean 52.2 mo., range 6–148.6 mo.). During surveillance, 46 (34.8%) and 19 (14.4%) of the patients experienced recurrence and progression at a median time of 14.1 mo. and 33.9 mo., respectively. The overall 5-year recurrence-free survival (RFS) and progression-free survival (PFS) rates were 73.4% and 88.4%, respectively.

**Figure 4:**
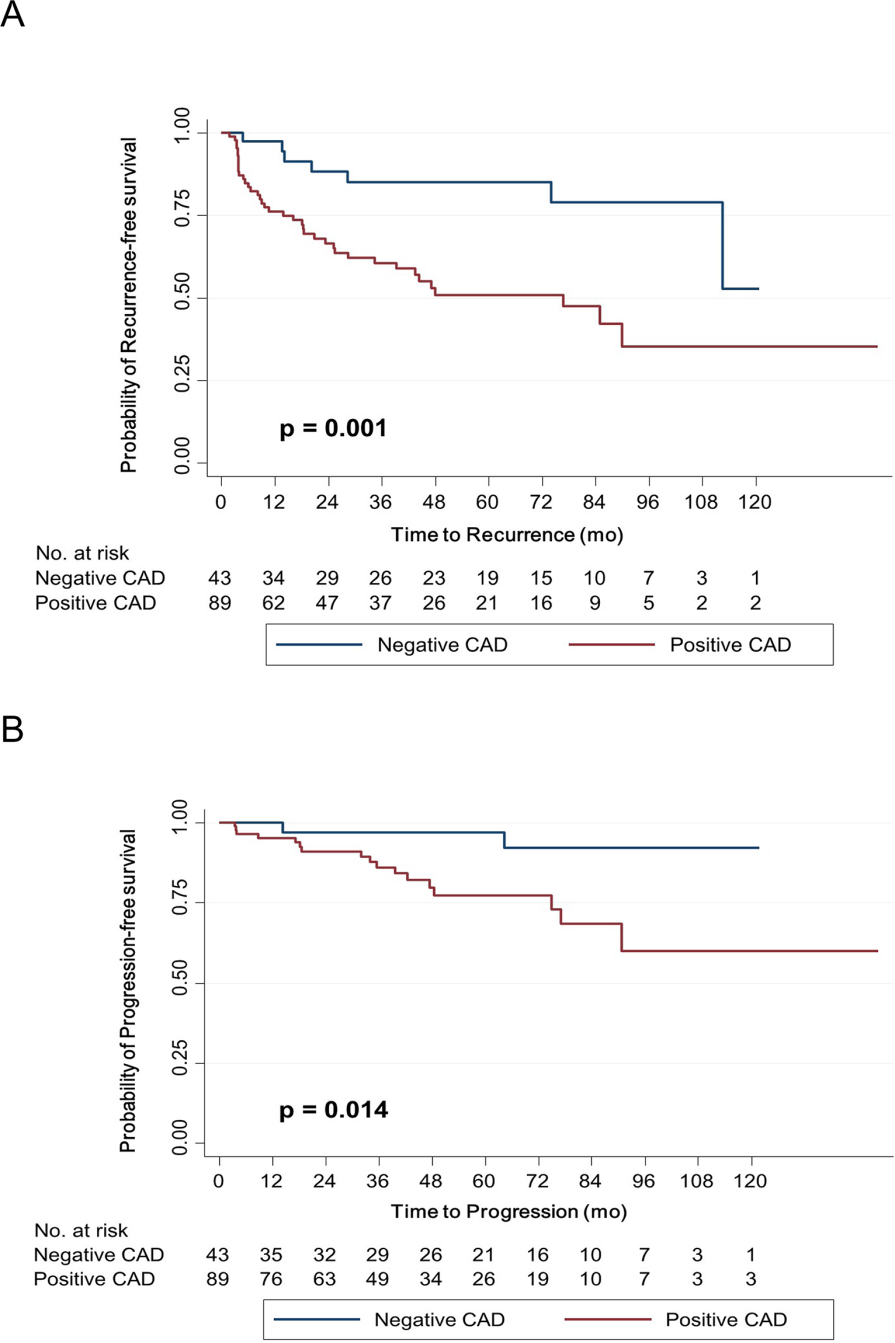
Kaplan–Meier survival curves for recurrence-free survival A. and progression-free survival B. according to caldesmon (CAD) expression Expression was dichotomized appropriately according to the immunohistochemical score, which was based on staining area and intensity.

In the Kaplan–Meier survival analysis, positive CAD expression was significantly associated with lower RFS (*p* = 0.001) and PFS (*p* = 0.014) rates than negative expression (Figure 4A and 4B). Similarly, univariate Cox regression analyses showed that CAD expression was a significant predictor for tumor recurrence and progression (Table 3). Compared with negative expression, positive expression of CAD was associated with a 3.499-fold (95% confidence interval, 1.557–7.865) and 5.255-fold (95% confidence interval, 1.207–22.878) increased risk for tumor recurrence (*p* = 0.002) and progression (*p* = 0.027), respectively.

**Table 3:** Univariate and multivariate Cox regression analyses of multiple variables for recurrence- and progression-free survival

Variables	Recurrence-free survival			Progression-free survival		
	HR	95% CI	*p*	HR	95% CI	*p*
**Univariate analysis**						
Age (as continuous variable)	1.023	0.998–1.049	0.069	1.045	1.001–1.091	0.043
Gender (male vs female)	0.966	0.465–2.005	0.925	1.687	0.639–4.449	0.291
Tumor size (<3 cm vs ≥3 cm)	2.080	1.155–3.744	0.015	1.859	0.741–4.664	0.186
Multifocality (single vs multiple)	2.106	1.146–3.870	0.016	1.272	0.458–3.536	0.645
Concomitant carcinoma *in situ* (no vs yes)	1.242	0.598–2.584	0.561	4.842	1.903–12.320	0.001
Morphology (papillary vs non-papillary)	0.883	0.346–2.255	0.795	2.592	0.824–8.155	0.103
Lymphovascular invasion (no vs yes)	1.447	0.717–2.919	0.302	1.837	0.658–5.125	0.246
Intravesical therapy (no vs yes)	0.684	0.378–1.238	0.210	0.375	0.135–1.043	0.060
T stage (Ta vs T1)	3.154	1.557–6.389	0.001	2.431	0.868–6.810	0.091
Grade (low vs high)	2.276	1.254–4.128	0.007	2.505	0.981–6.395	0.055
Caldesmon expression* (negative vs positive)	3.499	1.557–7.865	0.002	5.255	1.207–22.878	0.027
**Multivariate analysis**						
Age (as continuous variable)	1.016	0.989–1.045	0.246	1.022	0.973–1.074	0.380
Tumor size (<3 cm vs ≥3 cm)	1.475	0.755–2.883	0.255	1.558	0.540–4.494	0.412
Multifocality (single vs multiple)	4.148	1.889–9.109	<0.001	2.755	0.702–10.816	0.146
Concomitant carcinoma *in situ* (no vs yes)	0.498	0.214–1.158	0.105	4.688	1.290–17.042	0.019
Intravesical therapy (no vs yes)	0.221	0.096–0.506	<0.001	0.175	0.040–0.768	0.021
T stage (Ta vs T1)	2.141	0.816–5.618	0.122	0.694	0.167–2.876	0.615
Grade (low vs high)	2.536	1.214–5.296	0.013	1.208	0.312–4.673	0.785
Caldesmon expression* (negative vs positive)	1.656	0.591–4.644	0.337	5.983	1.165–30.736	0.032

* The immunohistochemical score was based on both staining area and intensity, and caldesmon expression was negative in 43 patients (32.6%), mild in 35 patients (26.5%), moderate in 37 patients (28.0%), and strong in 17 patients (12.9%). Its expression was dichotomized (negative vs. positive) because such grouping showed the most significant survival difference in the Kaplan–Meier analysis.

In multivariate analysis adjusting for CAD expression and multiple clinicopathological factors, CAD expression remained a significant predictor of tumor progression (Table 3). Patients with positive CAD expression were associated with a 5.983-fold (95% confidence interval, 1.165–30.736) increased risk for tumor progression compared with those with negative expression (*p* = 0.032). To externally validate the prognostic value of CAD expression, we analyzed the prognostic value of CAD in an independent cDNA microarray database including primary NMIBCs from Chungbuk National University [7]. We confirmed a significant difference in PFS according to CAD expression (Supplementary Figure S1).

### Migration and invasion of BC cells following silencing and overexpression of L-CAD

**Figure 5:**
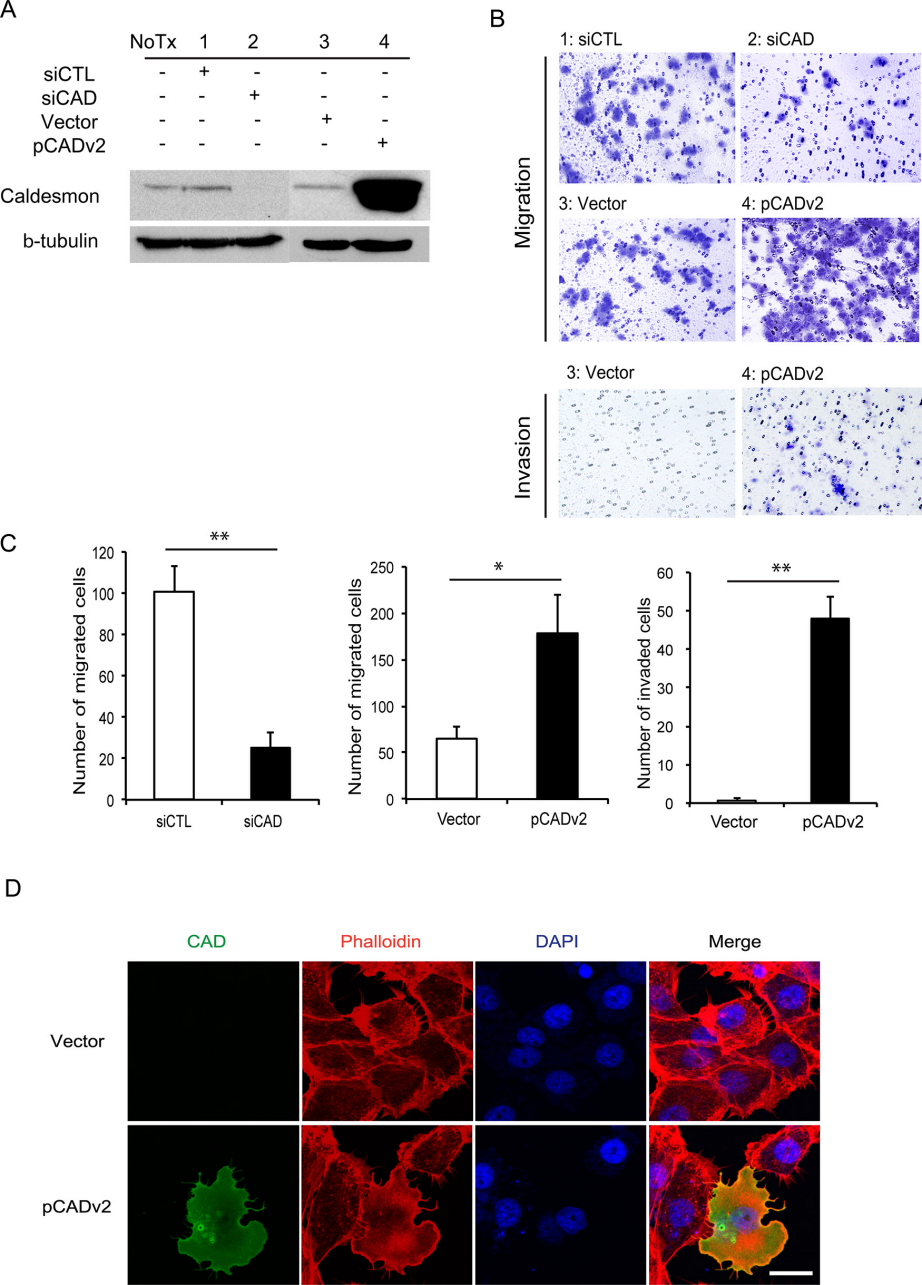
Migration and invasiveness of high-risk superficial bladder cancer (BC) cells following modulation of caldesmon (CAD) expression **A.** Western blot analyses of CAD are shown in 5637 BC cells transfected with CAD-specific small interfering RNA (siCAD) and scrambled sequence control siRNA (siCTL) or vector-overexpressing exogenous CAD transcript variant 2 (pCADv2) and its control vector (Vector). **B.** and **C.** Migration and invasion of 5637 cells after knockdown and overexpression of CAD are shown. (B) Representative images are shown from 3 independent experiments. The migrating and invading cells were examined using a Nikon Eclipse E400 microscope at 200× magnification. (C) The average number of migrating and invading cells per field of view (200×) was determined for each experimental group. The data were obtained from 5 randomly selected fields and the mean values are shown (*p < 0.05, **p < 0.01). **D.** Fluorescent phalloidin staining for actin in 5637 BC cells following transfection with the pCADv2 overexpression vector. Regular intracellular distributions of actin were noted in 5637 BC cells (upper column), whereas irregular patterns of cellular morphology and cytoskeletal redistribution were observed in CAD-overexpressing cells (lower column). CAD, actin cytoskeleton, and DAPI were detected as green, red, and blue fluorescence, respectively. Scale bar = 20 μm.

Based on our experiments, H-CAD or HeLa L-CAD was not detected in BC cell lines. Among WI-38 L-CAD, the major product was WI-38 L CAD II (transcript variant 2). Migration and invasion are hallmarks of tumor cells with invasive properties. As L-CAD is involved in cytoskeletal architecture and dynamics, we decreased the endogenous L-CAD protein expression in 5637 BC cells to determine whether L-CAD regulates migration and invasion of the cells. A significant decrease in the endogenous L-CAD protein level was observed in cells transfected with CAD-specific siRNA compared with those treated with vehicle or scrambled siRNA (Figure 5A). We found that silencing of L-CAD expression caused significant attenuation of cell migration and invasiveness (Figure 5B and 5C). Similarly, ectopic expression of L-CAD in 5637 cells by transfection with CALD1 transcript variant 2 (pCADv2) clearly augmented cell migration and invasive activity (Figure 5B and 5C). Neither silencing nor overexpression of CAD had a significant effect on the proliferative ability of 5637 cells in a WST-1 proliferation assay (Supplementary Figure S2). Changes in cytoskeletal morphology were also examined after transfection of pCADv2 into 5637 cells (Figure 5D). Dramatic reorganization of the actin cytoskeleton was observed in L-CAD-overexpressing 5637 cells. Loss of cell-cell adhesions was accompanied by the redistribution of actin filaments, indicating high motility of the cells.

## DISCUSSION

To our knowledge, this is the first study that demonstrates the expression, prognostic value, and potential functional mechanism of CAD in urothelial BC. Consistent with the AbM results, CAD expression, as determined by IHC, in muscle-invasive BC cells was significantly higher than that in NMIBC cells. Although AbM profiling showed that CAD expression in normal urothelial tissue was significantly higher than in BC tissues, this finding was not supported by IHC, i.e., CAD expression in normal cells was absent or very weak. Higher CAD expression in normal tissue in the AbM compared to BC tissue was thought to be due to abundant H-CAD of smooth muscle in normal bladder tissue. As shown in a schematic summary of CAD transcript variants (Figure 3A), the entire L-CAD sequence is included in the H-CAD sequence. Thus, the antibody used in our study detects both H-CAD and L-CAD. In our western blot and RT-PCR analyses to clarify the CAD isoform expressed in normal urothelial tissue, H-CAD was abundantly expressed in normal tissues, whereas H-CAD was rarely expressed in NMIBC tissues (Supplementary Figure S3). In contrast, NMIBC tissues and BC cell lines predominantly expressed L-CAD, while they rarely expressed H-CAD. Consistent with our finding, bladder smooth muscle cells showed relatively strong H-CAD expression, reaching 100% sensitivity and specificity, in a recent study [20]. After confirming the CAD isoforms expressed in normal tissue and BC, we focused on the NMIBC tissues and BC cells in subsequent experiments.

In the western blot analysis and IFA, we found variable CAD expression in four BC cell lines that represent different molecular features and clinical stages. While the RT4 cell line represents a well-differentiated, non-invasive, papillary tumor phenotype [21], the 5637 cell line is an *in vitro* model for high-risk NMIBC [22, 23], and T24 and TCC-SUP are invasive and metastatic BC cell lines, respectively [24]. The findings that BC cell lines with higher invasive potential displayed higher CAD expression in western blotting (Figure 2B) and IFA (Figure 2C) are consistent with the AbM and IHC findings. Taken together, these results confirm differential CAD expression according to the aggressiveness and invasive potential of BC cells.

Existing evidence supports the role of cytoskeletal proteins as candidates responsible for cancer progression [25–27]. In this context, CAD, which links myosin and actin filaments, seems to be implicated in the progression of several solid cancers. However, published studies regarding the function of CAD in cancer progression are conflicting. For example, CAD is reported to be associated with lymph node metastasis and poorer prognosis in oral squamous cell carcinoma [11] and upregulated L-CAD expression is associated with increased metastatic property and decreased susceptibility to chemoradiation therapy in colorectal cancer cells [12]. In contrast, CAD was reported to suppress cancer cell invasion by regulating podosome/invadopodium formation in colon and breast cancer cell lines [13] or by regulating phosphorylation through type I cGMP-dependent protein kinase in breast cancer cells [14]. Similarly, CAD expression in gastric cancer was lower in lymph node metastatic tissue compared to primary gastric cancer tissue, while overexpression of CAD in a lymph node metastatic gastric cancer cell line led to decrease in cell migration and invasion [15]. Conflicting results among studies may be partly attributable to different functions of CAD isoforms depending on CALD1 splice variants, although the precise reasons remain unclear.

Remarkably, no studies have investigated CAD protein expression and its prognostic value in BC. Our western blot and RT-PCR results indicate that BC cells do express L-CAD, specifically that encoded by WI-38 L-CAD II (transcript variant 2) (Figure 3). Furthermore, our immunohistochemical analyses in an independent validation cohort highlight the prognostic value of CAD in primary NMIBC. To date, only one study has suggested the CALD1 gene as a BC-associated candidate [28]. In a study based on axon array and RT-PCR analyses [28], a BC-specific splice variant of the CALD1 gene showed significant differential expression between BC and normal bladder mucosa. Most of the few studies on bladder tumors of smooth muscle origin did not specify these CAD isoforms [17, 18], or examined only H-CAD in bladder tumors originating from smooth muscle of stromal tissue [19, 20, 29].

Our findings from *in vitro* silencing and overexpression studies indicate that CAD promotes 5637 cell migration and invasiveness. We found some limitations in examining the effects of CAD in T24 and TCC-SUP cells. For example, although CAD-specific siRNA caused decrease in endogenous CAD expression in western blot analysis, high amounts of CAD were still detected in the transfected cells by IFA (Supplementary Figure S4). We also could not detect significant changes in CAD expression after transfection of pCADv2, probably due to constitutively high endogenous CAD expression in the two BC cell lines (Supplementary Figure S4). Thus, those two BC cell lines might not be appropriate models for analyzing the biological significance of CAD under our experimental conditions. After L-CAD overexpression, 5637 BC cells showed structural reorganization in their actin cytoskeleton. Considering the function of CAD in regulating the dynamics of the actin cytoskeleton, L-CAD isoform overexpression in BC may reflect dysfunctional upregulation of the cytoskeleton. Similar to our findings, recent studies have shown that L-CAD is crucially involved in microfilament network regulation, and acts as an important modulator of various cell functions, specifically cell motility [30, 31]. Proliferation of 5637 BC cells was not significantly different after L-CAD modulation (Supplementary Figure S2). These findings suggest that a potential mechanism underlying the role of CAD in BC mainly involves regulation of migration and invasive processes rather than proliferation of BC cells.

Given that the prognosis of tumors that progress from NMIBC to muscle-invasive BC is significantly worse than that of primary muscle-invasive BC [32, 33], immunohistochemical examination of L-CAD expression would be clinically useful for predicting tumor progression in patients with primary NMIBC. Another external validation from an independent cDNA microarray database supports the prognostic value of CAD (Supplementary Figure S1).

In conclusion, our findings collectively suggest that L-CAD overexpression in primary NMIBC is significantly associated with adverse pathological characteristics and tumor progression. Furthermore, these results indicate the clinical importance of L-CAD as a potential therapeutic target in BC. A possible mechanism of action of L-CAD is implicated in increases in cell motility and invasive characteristics through morphological changes in BC cells.

## MATERIALS AND METHODS

In the present study, we focused on urothelial carcinomas, hereafter synonymously called BC, and excluded other BC histologic variants (squamous, micropapillary, sarcomatoid, small cell, and adenocarcinoma).

### Study protocol

The study protocol was approved by the Institutional Review Board of the Eulji University Hospital (Approval No. 11–0081), and informed consent was obtained in all cases. Tumors were staged and graded according to the 7th American Joint Committee on Cancer criteria and 2004 World Health Organization grading system [34]. All TURBT surgeries were performed with a curative intent using a standard technique as previously described [35].

### AbM profiling of human bladder tissues

For the AbM analysis, a total of 11 BC tissue samples were acquired from patients who were diagnosed with primary NMIBC following complete TURBT (*n* = 5) and muscle-invasive BC following radical cystectomy (*n* = 6). A total of 7 normal bladder mucosal tissues were also obtained from patients undergoing TURBT (*n* = 3, from patients without concomitant carcinoma *in situ*) or from a tissue biobank (*n* = 4, tissues from patients undergoing transurethral resection of prostate and augmentation cystoplasty) of the Eulji University Hospital, a member of the National Biobank of Korea. After the tissue samples were obtained intraoperatively, they were immediately frozen in liquid nitrogen and stored at −70°C until protein extraction. Protein profiling of 18 tissue samples was performed and analyzed using AbM assay kits with 656 antibodies (Fullmoon Biosystems, Sunnyvale, CA), as described in our previous study [9]. The numerical data were analyzed using Genowiz 4.0 (Ocimum Biosolutions, Hyderabad, India). Global normalization was used for data analysis. A 2-class *t*-test or paired *t*-test was performed to compare each experiment-reference group combination. The averages of normalized ratios were calculated by dividing the average normalized BC sample intensity by the average normalized normal urothelium sample intensity. The benchmarks for upregulation and downregulation were based on both fold-change criteria (>1.5-fold or <0.667-fold) and statistical significance (*p* < 0.1). After analysis, the data were annotated using protein information in the UniProt database. A hierarchical clustering algorithm was used to evaluate the association of protein profiles and the study individuals, with Pearson correlation as the distance metric (where distance = (1 − Pearson correlation value)/2), and the average linkage method, as described previously [36]. The array data were deposited in the NCBI's Gene Expression Omnibus and are accessible through GEO Series accession number GSE69736.

### Cell culture and transfection

The HeLa S3 cell line, WI-38 cell line, and BC cell lines (5637, RT4, T24, and TCC-SUP) were obtained from the Korean Cell Line Bank (Seoul, South Korea) and American Type Culture Collection (ATCC, Manassas, VA). HeLa S3, WI-38, and 5637 cells were cultured in RPMI 1640 (Lonza, Walkersville, MD) supplemented with 10% fetal bovine serum (FBS, Gibco BRL, Grand Island, NY) and 1% penicillin/streptomycin (HyClone, GE Healthcare, Little Chalfont, UK). RT4 and T24 cells were cultured in Dulbecco's Modified Eagle's Medium (Welgene, Daegu, South Korea), and TCC-SUP cells were cultured in Eagle's Minimal Essential Medium (ATCC). The cells were maintained in a humidified atmosphere of 5% CO_2_ at 37°C. Accutarget predesigned siRNAs specific for human CAD and scramble siRNAs purchased from Bioneer (Daejeon, South Korea) were used for knockdown of CAD expression. A plasmid expressing CAD transcript variant 2 (#EX-Z0014-M68, designated as ‘pCADv2’) and the blank vector (#EX-NEG-M68) were purchased from GeneCopoeia (Rockville, MD). Transfections were performed using Lipofectamine 3000 or Lipofectamine RNAiMAX transfection reagent, according to the manufacturer's instructions (Invitrogen, Carlsbad, CA).

### Western blot

Whole cell proteins were isolated using 1 × SDS buffer containing 62.5 mM Tris-HCL at pH 6.8, 2% w/v SDS, 10% v/v glycerol, 50 mM dithiothreitol, and 0.01% w/v bromophenol blue. The cell suspension was boiled for 10 min and then centrifuged at 13,000 rpm for 8 min. The proteins were resolved by electrophoresis in a 12% SDS-polyacrylamide gel and transferred onto a nitrocellulose membrane (GE Healthcare). The membranes were blocked with 5% skim milk in Tris-buffered saline with 0.1% Tween 20 (TBST). Polyclonal rabbit anti-caldesmon (Sigma-Aldrich, St. Louis, MO) and mouse anti-β-tubulin (Sigma-Aldrich) were used as primary antibodies. HRP-conjugated anti-rabbit or anti-mouse antibodies (Santa Cruz Biotechnology, Dallas, TX) were used as secondary antibodies. The results were visualized using an ECL detection reagent (Santa Cruz Biotechnology).

### Immunofluorescence assay (IFA)

Each cell line was seeded onto microscopy cover glasses in 24-well tissue culture plates at a density of 1 × 10^5^ cells/well. After overnight culture, the culture medium was removed, and the cells were washed with PBS and fixed with 4% paraformaldehyde in PBS for 15 min at room temperature. The fixed cells were permeabilized with 0.25% Triton X-100 for 15 min. After blocking in PBS containing 3% bovine serum albumin for 30 min at 4°C, cells were incubated overnight at 4°C with the polyclonal rabbit anti-caldesmon antibody. After washing with TBST, samples were incubated for 15 min with AlexaFluor 488-conjugated goat anti-rabbit antibody (Invitrogen), washed again, and actin-stained with AlexaFluor 568-conjugated phalloidin (Invitrogen). After washing, each slide was incubated with 4′,6-diamidino-2-phenylindole (DAPI) to visualize nuclei. Samples were mounted in Gel/Mount Reagent (Biomeda, Foster City, CA). Images were obtained using a Nikon ECLIPS E400 microscope (Nikon Instruments, Melville, NY) or ZEISS LSM 780 (Carl Zeiss, Oberkochen, Germany). Images were analyzed using Nikon NIS Element F Microscope Imaging or ZEN 2012 Software.

### RT-PCR

Total RNAs were isolated using an easy-BLUE (iNtRon Biotechnology, Sungnam, South Korea) total RNA isolation kit. Total RNA was reverse transcribed using the ReverTra Ace first-strand cDNA synthesis system (Toyobo, Osaka, Japan), according to the manufacturer's instructions. PCR was performed using a DiaStar Taq DNA polymerase kit (SolGent, Daejeon, South Korea). The cycling conditions were as follows: 95°C for 2 min and 35 cycles of 94°C for 20 s, 56°C for 40 s, 72°C for 90 s. The primers were synthesized by GENOTECH (Daejeon, South Korea) and their sequences were as follows: Pm, 5′-GTT TAA GTT TGT GGG TCA TGA ATT CTC C-3′; Pn1, 5′-ATG CTG GGT GGA TCC GGA TC-3′; Pn2, 5′-ATG GAT GAT TTT GAG CGT CG-3′; H-CAD S, 5′-GGA GGA AGA GAA GGC TAA G-3′; H-CAD AS, 5′-TTT GAA GGT AGG CTT GTC T-3′. Amplicons were analyzed by gel electrophoresis in 2% agarose gels with 0.5 μg/mL ethidium bromide and gel images were acquired using a Gel Doc XR+ imaging system (Bio-Rad, Hercules, CA).

### Migration and invasion assay

The migration and invasion assay was performed using the CytoSelect 24-Well Cell Migration and Invasion Assay kit (8 μm, Colorimetric Format, Cell Biolabs, San Diego, CA), according to the manufacturer's instructions. Briefly, the migration assay plate was incubated at room temperature for 10 min, and an invasion basement membrane layer of the cell culture insert was rehydrated with warm serum-free RPMI 1640 media for 1 h at room temperature. Cells were detached from the culture plate by trypsinization and resuspended in serum-free RPMI 1640 media. RPMI 1640 media containing 10% FBS was added into the lower chamber, and the cell suspensions were applied to the upper chamber of the migration/invasion insert. Chambers were incubated at 37°C for 24 h and the membrane was stained with cell stain solution at room temperature. The stained migratory or invasive cells were analyzed by a Nikon ECLIPS TS100 inverted microscope (Nikon Instruments).

### Cell proliferation assay

The amount of viable cells was measured using the WST-1 cell proliferation reagent (Roche Applied Sciences, Indianapolis, IN). Equal numbers of cells were seeded on 96-well culture plates and cultured for 24 and 48 h, and then the culture media were replaced by media containing WST-1 (1:10 dilution) and incubated for 30 min at 37°C in a cell incubator. The optical density was measured at 450 nm and 650 nm.

### Pathology evaluation and IHC

To confirm the expression pattern of CAD in the AbM profiling, IHC was performed on tissue specimens, including NMIBC, muscle-invasive BC, and normal urothelium. In addition, to validate the prognostic values of CAD in primary NMIBC, immunohistochemical analysis was performed in an independent primary NMIBC cohort comprising 132 patients at the Eulji University Hospital. The patients in the validation cohort underwent surveillance according to our follow-up protocol [37, 38]. Patients with short-term follow-up periods (less than 6 months) were excluded. All pathology slides were thoroughly re-evaluated by a single uropathologist (JHK).

Immunohistochemical staining and evaluation was performed as described in our previous studies [9, 38], using a polyclonal rabbit anti-caldesmon antibody (Sigma-Aldrich). The optimal primary antibody dilution was predetermined using appropriate positive control tissue (uterine leiomyoma) and negative control (omission of primary antibody). Immunoreactivity was evaluated by light microscopy twice, at 4-week intervals, by a single uropathologist (JHK) who was blinded to clinical outcomes. A repeat reading of the same sample showed high concordance (κ = 0.864, *p* < 0.001). Immunoreactivity was evaluated semiquantitatively by integrating the staining intensity (0–3) and percentage of positively stained cells (1, ≤24%; 2, 25%–49%; 3, 50%–74%; 4, ≥75%) [9, 38]. An immunohistochemical score was calculated by multiplying the intensity scores and staining area, and was classified as representing negative (0–1), mild (2–3), moderate (4–8), or strong (9–12) expression.

### Statistical analysis

Chi-square tests were used to evaluate the association between categorical variables. Student's *t*-tests were used to compare continuous variables in the AbM and migration/invasion assay. In a validation cohort, RFS and PFS rates were analyzed. Tumor recurrence was defined as the presence of pathological evidence of similar- or lower-stage disease by bladder biopsy or TURBT, and progression was defined as a pathological shift to more advanced stage disease. The Kaplan–Meier method was used to calculate RFS and PFS rates, and differences were evaluated with the log-rank test.

For analysis, CAD expression based on the immunohistochemical score was dichotomized appropriately because such grouping showed the most significant survival difference in Kaplan–Meier analysis. The prognostic significance of CAD expression was assessed by univariate and multivariate Cox regression analysis models. All tests were two-sided, with *p* < 0.05 considered significant. All statistical analyses were performed using Stata/SE software, version 12.1 (Stata Corporation, College Station, TX).

## SUPPLEMENTARY FIGURES


