# Supplementary material for: Overexpression of caldesmon is associated with tumor progression in patients with primary non-muscle-invasive bladder cancer
Source: Oncotarget. 2015 Sep 25;6(37):40370–84. doi: 10.18632/oncotarget.5458 (PMC4741901; doi:10.18632/oncotarget.5458)
Supplement: Supplementary file 1 [file oncotarget-06-40370-s001.pdf]

SUPPLEMENTARY FIGURES

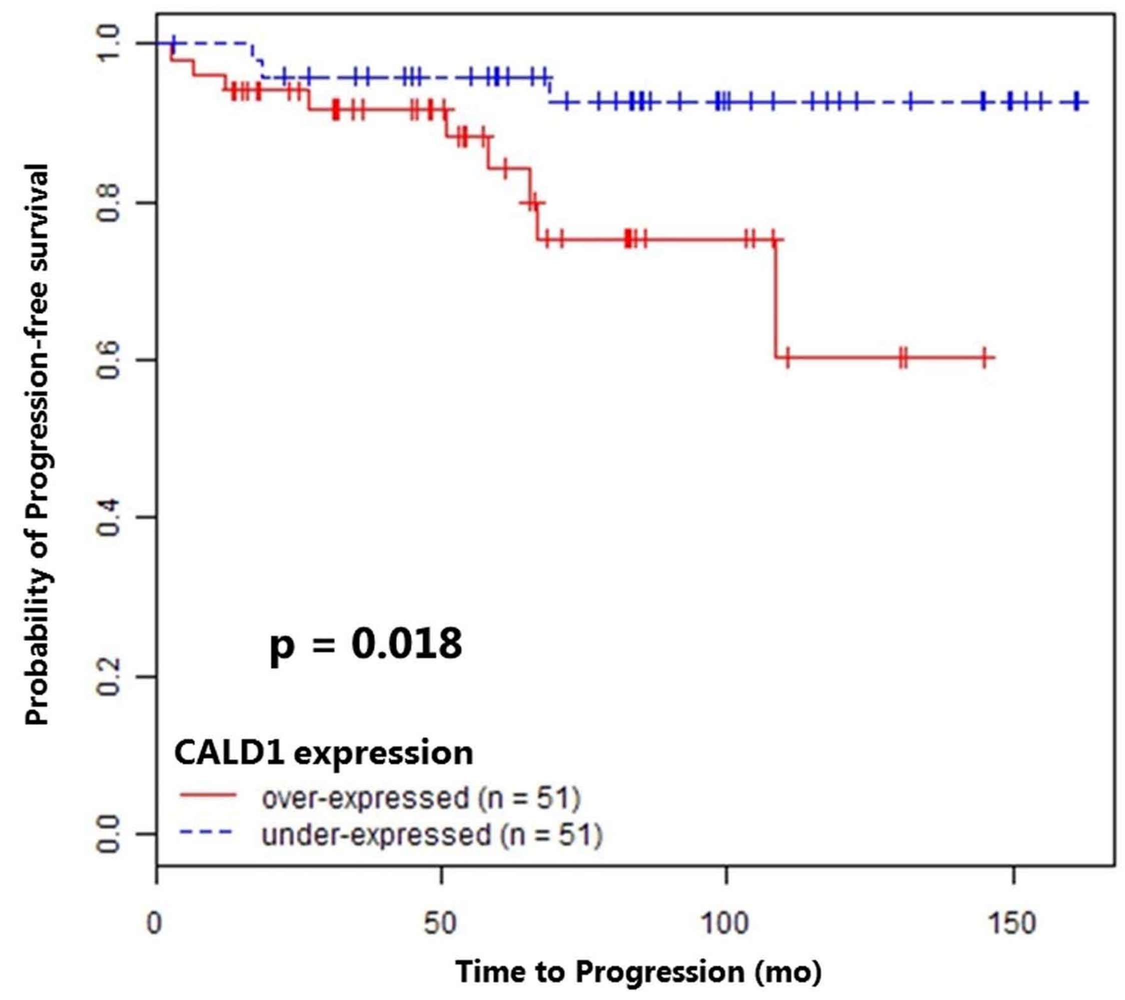

Supplementary Figure S1: Kaplan–Meier survival curves for progression-free survival according to caldesmon mRNA expression in an independent cDNA microarray database including primary non-muscle-invasive bladder cancer.

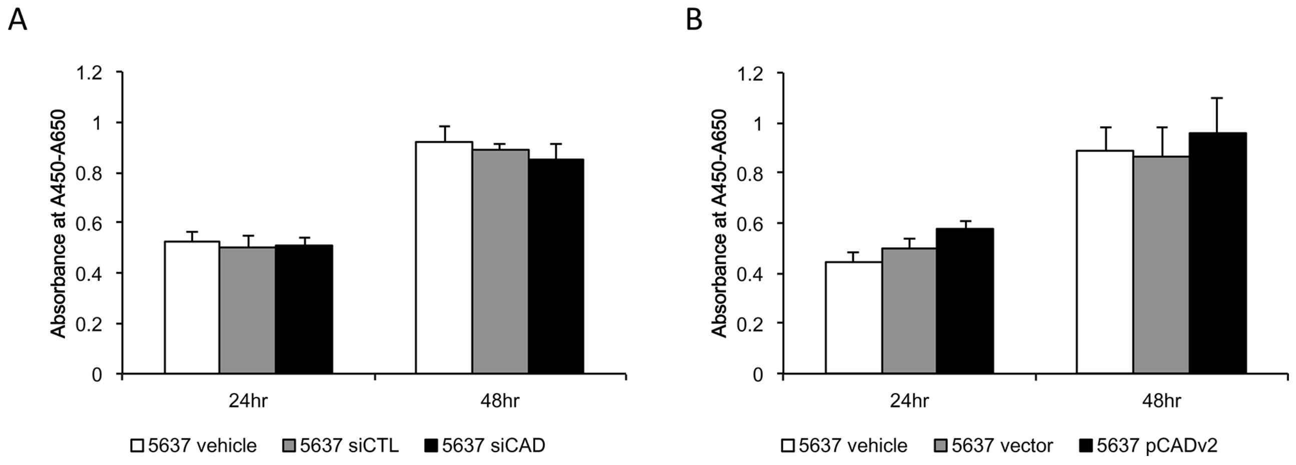

Supplementary Figure S2: Proliferation of bladder cancer (BC) cells after knockdown A. and overexpression B. of caldesmon (CAD) in the 5637 cell line. The proliferation of BC cells was analyzed by WST-1 assay. The results represent the means of triplicate experiments, and standard deviations are indicated with error bars. No significant differences in proliferation of BC cells were observed after transfection with CAD-specific small interfering RNA (siCAD) and scrambled sequence control siRNA (siCTL) or vector-overexpressing exogenous CAD transcript variant 2 (pCADv2) and its control vector (Vector), respectively.

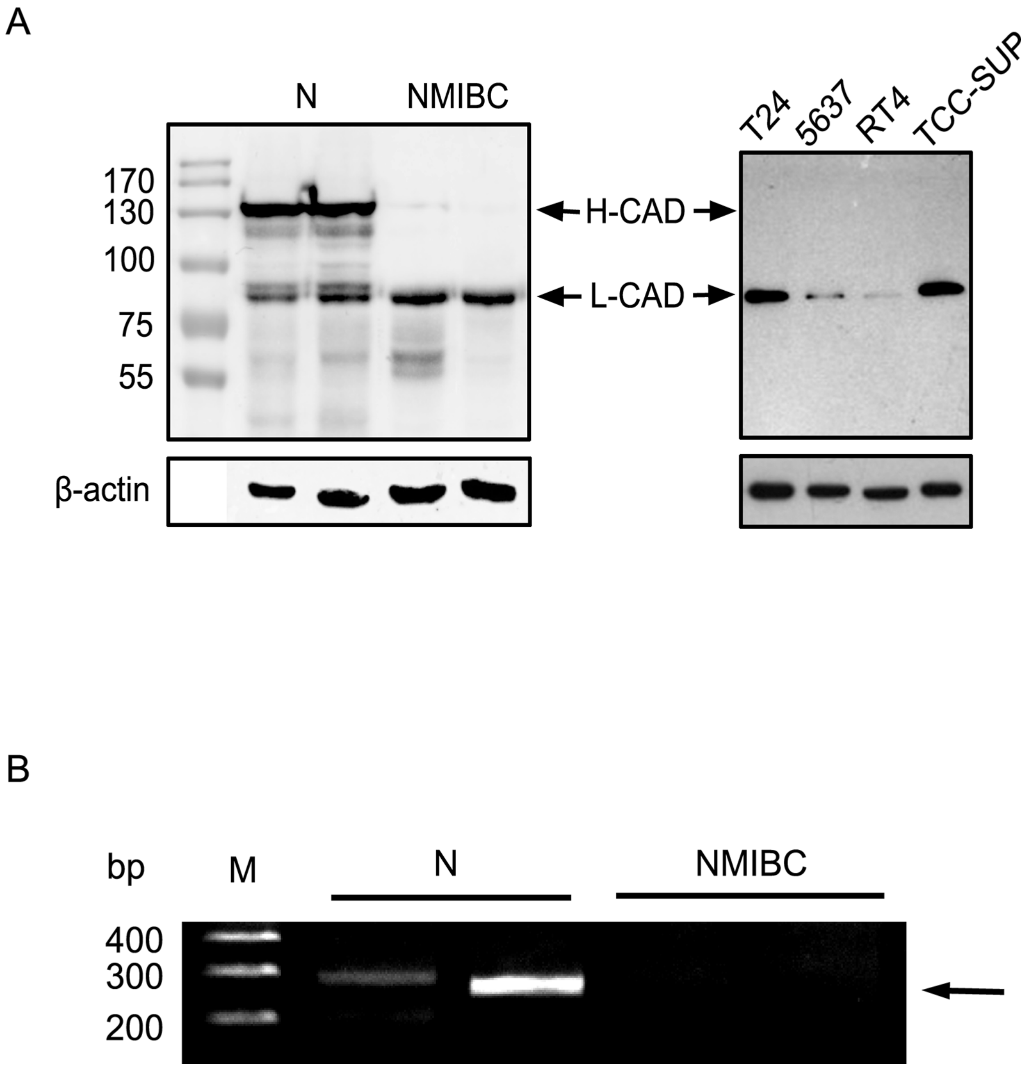

Supplementary Figure S3: Expression of caldesmon (CAD) isoforms in human bladder tissues and bladder cancer (BC) cell lines. Two normal bladder mucosa (designated as 'N') tissues and two non-muscle-invasive BC (NMIBC) tissues were obtained from 4 different patients, and their expression of CAD isoforms was analyzed. **A.** Western blot analysis of CAD isoforms in human bladder tissues and BC cell lines. The expected size of H-CAD and L-CAD in western blot analysis is indicated by arrows. **B.** RT-PCR analysis of H-CAD in normal bladder mucosa and NMIBC tissues. The expected amplified PCR band with H-CAD-specific primers is indicated by an arrow.

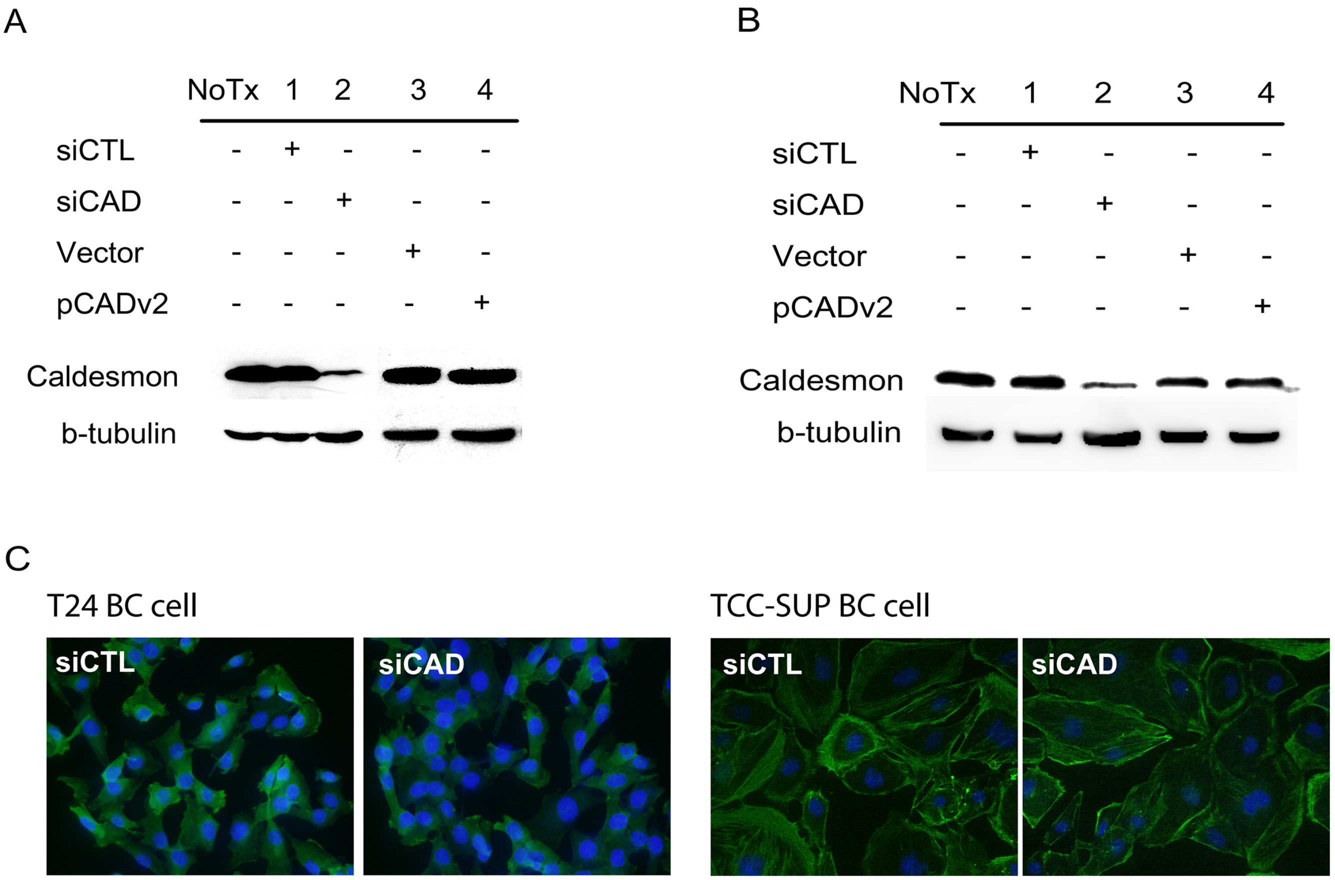

Supplementary Figure S4: Caldesmon (CAD) expression in T24 and TCC-SUP cell lines following knockdown and overexpression of CAD. Western blot analysis of CAD is shown in T24 cells **A.** and TCC-SUP cells **B.** after transfection with CAD-specific small interfering RNA (siCAD) and scrambled sequence control siRNA (siCTL) or vector-overexpressing exogenous CAD transcript variant 2 (pCADv2) and its control vector (Vector), respectively. **C.** Immunofluorescence assay for CAD in T24 and TCC-SUP cells after transfection with siCTL and siCAD. CAD and DAPI were detected by green and blue fluorescence, respectively (400× magnification).
